# Microbial composition and diversity of the tobacco leaf phyllosphere during plant development

**DOI:** 10.3389/fmicb.2023.1199241

**Published:** 2023-07-12

**Authors:** Jianing Gao, Ernest Uwiringiyimana, Dan Zhang

**Affiliations:** ^1^College of Tourism and Geographical Science, Leshan Normal University, Leshan, China; ^2^Institute of Soil Science, Chinese Academy of Sciences, Nanjing, China; ^3^University of Chinese Academy of Sciences, Beijing, China; ^4^Key Laboratory of Mountain Surface Processes and Ecological Regulation, Institute of Mountain Hazards and Environment, Chinese Academy of Sciences, Chengdu, China

**Keywords:** tobacco, community dynamics, phyllosphere microbiota, leaf nutrients, growth stage

## Abstract

Phyllosphere-associated microorganisms affect host plant’s nutrients availability, its growth and ecological functions. Tobacco leaves provide a wide-area habitat for microbial life. Previous studies have mainly focused on phyllosphere microbiota at one time point of tobacco growth process, but more is unknown about dynamic changes in phyllospheric microbial composition from earlier to the late stage of plant development. In the current study, we had determined the bacterial and fungal communities succession of tobacco growth stages (i.e., seedling, squaring, and maturing) by using both 16S rRNA sequencing for bacterial and ITS sequencing for fungi. Our results demonstrated that among tobacco growth stages, the phyllospheric bacterial communities went through more distinct succession than the fungal communities did. Proteobacteria and Actinobacteria exerted the most influence in tobacco development from seedling to squaring stages. At maturing stage, Proteobacteria and Actinobacteria dominance was gradually replaced by Firmicutes and Bacteroidetes. Network analysis revealed that Proteobacteria, as the core phyllospheric microbia, played essential role in stabilizing the whole bacterial network during tobacco development, and consequently rendered it to more profound ecological functions. During tobacco development, the contents of leaf sugar, nicotine, nitrogen and potassium were significantly correlated with either bacterial or fungal communities, and these abiotic factors accounted for 39.3 and 51.5% of the total variation, respectively. We overall evinced that the development of tobacco phyllosphere is accompanied by variant dynamics of phyllospheric microbial community.

## 1. Introduction

In nature, plants and their associated microbes can establish a “holobiont” through symbiosis (Lyu et al., 2021). Healthy and symptomless infected plants integrate with hyperdiverse microorganisms like archaea, oomycetes, bacteria, fungi, and occasionally protozoa and nematodes, to form complex microecosystems and affect plant growth and productivity (Liu et al., 2020). These microbiotas in plants facilitate host nutrient acquisition, hormone production, and immunity stimulation (Bhatt et al., 2021). To sustain and utilize more of these benefits, plants modulate microbial assemblage with both the leaf or root surface (i.e., epiphytes) and the internal tissues (i.e., endophytes; Gong and Xin, 2021).

Among the plant’s aerial parts including stems, leaves, fruits, pollens and nectars, the leaves are identified as the ones with the most dominant tissue (Liu et al., 2020). In addition, the area of terrestrial plant leaves has been previously estimated at approximately 10^9^ km^2^ and support a tremendous diversity of microorganisms, of which bacteria is found to outweigh other populations in both cells number (average of 10^6^–10^7^ cells/cm^2^) and diversity of taxonomic groups. Apart from bacteria, the foliage is also inhabited by abundant yeasts and filamentous fungi with population ranging between 10 and 10^4^ cells/cm^2^. Microbial lineages that inhabit the leaf surface (commonly referred to as phyllosphere) have not received enough attention compared to their counterparts, foliar endophytic microbes. Therefore, the ecological functions of plant phyllosphere associated with microbial species are still ambiguous.

Unlike the endophytic microorganisms of foliage, phyllosphere tend to render a dynamic and open habitat for microbial life (Garg, 2021). The management of phyllosphere microbiota is a serious challenge due to the multiple factors that can influence the phyllosphere microbial community structure and assembly. The host plant often plays a role of filtering and favors specific taxa in the phyllosphere. Studies demonstrated that the leaf traits such as waxiness, age, wettability and chemical composition played a primary role in foliar colonization and assemblage of microorganisms (Agler et al., 2016). For instance, the microbes residing on leaves assemble to exploit inorganic nutrients, water and exudated sugars, and emit volatile organic compounds like methane and/or methanol (Bashir et al., 2022). Mercier and Lindow (2000) found that carbohydrates (particularly sugars), amino and organic acids which are secreted from the leaf surface render it to diverse habitat for microbial colonization. Environmental factors including temperature, humidity and solar radiation levels, geographical location, and pollutants including particulate matter, pesticides, herbicides, toxic metals can all determine phyllospheric microbial community structure.

Tobacco (*Nicotiana tabacum* L.) has received considerable attention as one of the world’s most profitable cash crops grown widely (Zhao et al., 2007). Leaf represents the most, if not the only, important part in tobacco productivity, and is the wide-area habitat abounding with microbial life. The tobacco phyllosphere microbiota has recently been studied extensively. For instance, Wang et al. (2022) demonstrated the variation of tobacco’s phyllospheric microbial community as a result of the change in summer climatic variables and wildfire disease. The recent study also showed how tobacco leaf’s microorganism communities are affected by local environments (Hu et al., 2022). However, there is still more unknowns to be addressed. Plant development and/or age also can promote shifts in microbial communities, but very little was learned about the role of the phyllosphere microbiome and how they are pioneered and maintained. Therefore, understanding how tobacco development interacts with phyllosphere colonization is essential for comprehensive effect of phyllosphere microbiota on plant traits at ecological scales and evolutionary stages. Thus, in this study, we aimed to answer few important questions below:

1. How, dynamically, do bacterial and fungal communities alter during tobacco development?
2. How do co-occurrence network patterns and core microbial species differ between bacteria and fungi?
3. What are the key environmental factors influencing phyllospheric bacterial and fungal community succession? We hypothesized that tobacco phyllospheric microbia is a dynamic process determined by different microorganisms at different stages of plant development.

## 2. Materials and methods

### 2.1. General description of the study area

Our study was conducted at Tobacco Research Station of Panzhihua City (27.11° N, 102.15° E, 1608 m above sea level), one of the tobacco production areas in Sichuan Province. According to statistics of year 2019, the total output of tobacco leaves from Sichuan Province accounted for 160,400 tons, behind just Yunnan, Guizhou and Henan provinces; hence, one of the largest tobacco producing and exporting provinces in China. The local climate is featured by seasonal drought, with the average annual temperature of 19.7°C and 7,200°C cumulatively. Moreover, the duration of sunshine is 2,700 h annually. The average annual precipitation and evaporation are 1,100 mm and 2002.7 mm, respectively. In this area, the growing season for tobacco is from April to October. The area is characterized by sparse natural vegetation, and tobacco is the notable cash crop there.

### 2.2. Experimental design and leaf sampling

We established 18 research blocks in the study area, each with 6 m of length and 4 m of width. The total 18 blocks were laid out with 1 m apart of each other, and were linked with PVC pipes to establish the same conditions and agricultural management (i.e., foliar spray and tillage). Based on the traditional classification of tobacco development, the growth of *Nicotiana tabacum* was classified into three stages: seedling stage, squaring stage, and maturing stage. We conducted sampling during April (seedling stage), July (squaring stage) and October (maturing stage) in 2021, while ensuring that there was no rainfall 5 days before sampling. The phenotypic characteristics of plants at different stages were shown in Supplementary Figure S1. By minimizing the variation caused by external factors other than growth stage, we selected 6 plots during the sampling in seedling stage. Noting that the plots are farther from the extreme plots and their plants are growing well. Four to six seemingly healthy plants in each plot were selected to take leaves from. Then, those plants were adapted to be the reference for taking samples at each growth stage. One or two leaves from each plant per plot were collected and pooled into one composite sample. Thus, six biological replicates were taken from six different blocks at each stage, resulting in a total of 18 leaf samples. Thereafter, the sterile sealed polyethylene bags were used to pack the samples together and immediately taken to the laboratory with the ice boxes for pretreatment.

### 2.3. Leaf nutrient analysis and seasonal data

Tobacco leaves samples were dried at 105°C for 45 min, for enzymes deactivation, then placed in the blast dryer (70 ± 5°C) to ensure the complete dry. The dried samples were ground to pass through 0.25 mm sieve for chemical compositions analysis. Major elements including nitrogen (N) and potassium (K) concentrations were determined using H_2_SO_4_-H_2_O_2_ digestion by Kjeldahl and flame photometer methods, respectively. Other elements including calcium (Ca), magnesium (Mg) and chlorine (Cl) concentrations were determined using HNO_3_-HClO_4_ digestion by AAS method (iCE 3,000, Thermo). The content of nicotine in the samples were determined by silicotungstic acid gravimetric method (YC/T 247–2008). The extraction method of total sugar was referred from Mercier and Lindow (2000). The content levels were determined by high-performance liquid chromatography, with a Shodex NH2P-50 analytical column linked to an evaporative light-scattering detector (ELSD-16, Shimadzu).

The seasonal data were obtained from the Climate Research dataset for the period April to October of 2021, with grids of 0.5 resolution using the Climate explorer webpage.^1^ To this end, the climate data on monthly basis from the geographically nearest grid cell to the coordinates of our study area were downloaded. The climate variables used include: mean temperature (TMP), mean maximum temperature (TMX), mean minimum temperature (TMN), precipitation rate (PRE), vapor pressure (VAP) and potential evapo-transpiration (PET).

### 2.4. Samples treatment, DNA preparation and further sequencing

As stressed previously, the atmospheric dust can exist at the surfaces of leaves and act as an adulterant leading to confounding results along with phyllospheric microorganisms (Joshi, 2008). Hence, removal of unsettled microbes from leaf surface was performed by washing for all collected leaf samples and then followed the protocol from Redford and Fierer (2009).

The DNA extraction, amplification and sequencing, from samples, were performed at the Paisennuo Biotechnology Co., Shanghai, China. The extraction of DNA was done at 4°C indoors. The DNA was isolated from 0.5 g of the prepared pellets as above, using buffer solution. Quality of the extracted DNA, including the concentration and purity, was further detected by Nanodrop NC2000 spectrophotometer (Thermo Scientific). Considering the high-throughput Illumina sequencing, PCR amplification on both 16S rRNA for bacteria and ITS for fungi was carried out employing universal primer pairs as 341F/806R and ITS1F/ITS1R, respectively. The condition of PCR amplification was as below: pre-denaturation at 98°C for 2 min, denaturation for 15 s (25–30 cycles) at 98°C, annealing for 30s at 55°C, extension at 72°C for 30s, and then incubation at 72°C for 5 min. Subsequently, amplicons were further quantified and library was constructed based on the Illumina NovaSeq 6,000 platform. After sequencing, the raw data produced was archived into the NCBI database (MbPA2021091709 for bacteria and MbPL2021081633 for fungi).

### 2.5. Bioinformatics and statistical analysis

Sequences obtained from the above was subjected to bioinformatic analysis by using the QIIME pipeline. In order to get the annotation classification information of OTU, the typical sequences of 16S rRNA OTU and ITS OTU were compared by Silva_132 and Unite_8.0 reference sequence databases, respectively.

The alpha diversity of microorganisms was learned for each sample based on the Chao1, Observed_species, Goods_coverage, and Shannon and Simpson indices. Beta diversity was estimated using Bray-curtis distances. Non-metric multidimensional scaling analysis based on Bray-curtis dissimilarities among all samples was applied to summarize the patterns of bacterial and fungal community structures. LEfSe was employed to find the biomarker of each stage (Segata et al., 2011). Non-parametric KW sum-rank test was used to determine taxa with significant differential abundance, and Wilcoxon rank-sum test was then applied to evaluate dissimilarities between groups. The Linear discriminant analysis effect size (threshold value of 3.5) was employed to determine discriminative taxa, followed by community dissimilarities of microbiota exhibited by Cladogram diagrams. MetaCyc^2^ functional annotations of microbial communities were predicted using the PICRUSt2 method.

At last, we performed network analysis to tell the complexity of taxon-to-taxon interaction. First, only OTUs that were detected in 5 or more for all samples were retained for network analysis. Similarity matrix was calculated using the method of SparCC, and using random matrix theory to determine the filtering threshold of correlation value. Subsequently, using the R function “induced_subgraph” in the “igraph” package, the nodes with the top 100 average abundances were filtered to build network. The default display removed the negative correlation data of the associated current network to build a co-occurrence network with visualization by using the R package “ggraph.” Several basic networks for topologic properties were also determined, including average nearest neighbor degree, betweenness centrality, average path length, degree centralization, density, closeness centrality, degree assortativity, numbers of vertice and edge, and transitivity. Finally, according to the modular cutting results, we determined the role of each node based on the score values of within module connectivity (Zi) and between module connectivity (Pi). The above-mentioned analysis was explored by“genescloud” of Personalbio Co., Ltd. (Shanghai).^3^

The ANOVA was conducted using IBM SPSS 21.0 software and Duncan-test, *p* < 0.05 was used to analyze the differences of phytochemical estimates concentrations. The correlation between relative abundance of microbial community at the genus level and phytochemical parameters was determined using the Spearman correlation analysis.

## 3. Results

### 3.1. Alpha diversity of microbial communities

Sequencing efforts resulted in 1,809,586 (bacteria) and 2,274,495 (fungi) reads for 18 leaf samples. A total of 3,265 and 4,205 OTUs with 99% of similarity were obtained after quality filtering and chimera removal. Considering samples comparison, the reads number per sample was set to the minimum (i.e., 26,384 for bacteria and 75,907 for fungi), and a total of 2,616 and 2,346 OTUs were remained (Supplementary Table S1).

The Alpha diversity of both phyllospheric bacterial and fungal communities are summarized in Table 1. Observed_species and Chao 1 of both bacterial and fungal communities showed the same trend, and were significantly higher in the squaring stage than in the seedling and maturing stages as revealed from Duncan test. The Shannon and Simpson indices of microbial communities were not statistically significant different. Nevertheless, in both bacteria and fungi, the lowest Goods_coverage was recorded at squaring stage, particularly in bacterial community, which can also be characterized by the rank-abundance curve (Supplementary Figure S2). Compared to the fungal communities, the rank-abundances curves for bacteria decreased dramatically at the squaring stage, as a proof that the numbers of dominant bacteria in these samples were relatively high.

**Table 1:** Alpha diversity of phyllospheric bacterial and fungal communities.

Community	Growth stages	Chao1		Observed_species		Shannon		Simpson		Goods_coverage	
		Average	Duncan	Average	Duncan	Average	Duncan	Average	Duncan	Average	Duncan
Bacteria	Seedling	265.83	b	153	ab	5.85	a	0.95	a	0.8807	a
	Squaring	939.05	a	295	a	6.85	a	0.96	a	0.6561	b
	Maturing	103.79	b	95	b	5.82	a	0.97	a	0.9756	a
Fungi	Seedling	177.78	b	174	b	3.44	a	0.8	a	0.9998	a
	Squaring	375.85	a	369	a	3.68	a	0.65	a	0.9997	b
	Maturing	164.3	b	160	b	2.56	a	0.6	a	0.9998	ab

Different lowercase letters, column wise, represent that the values are significant different among growth stages at 0.05 level, following multiple comparisons with Duncan test.

### 3.2. Microbial community composition and bray-Curtis dissimilarities

**Figure 1:**
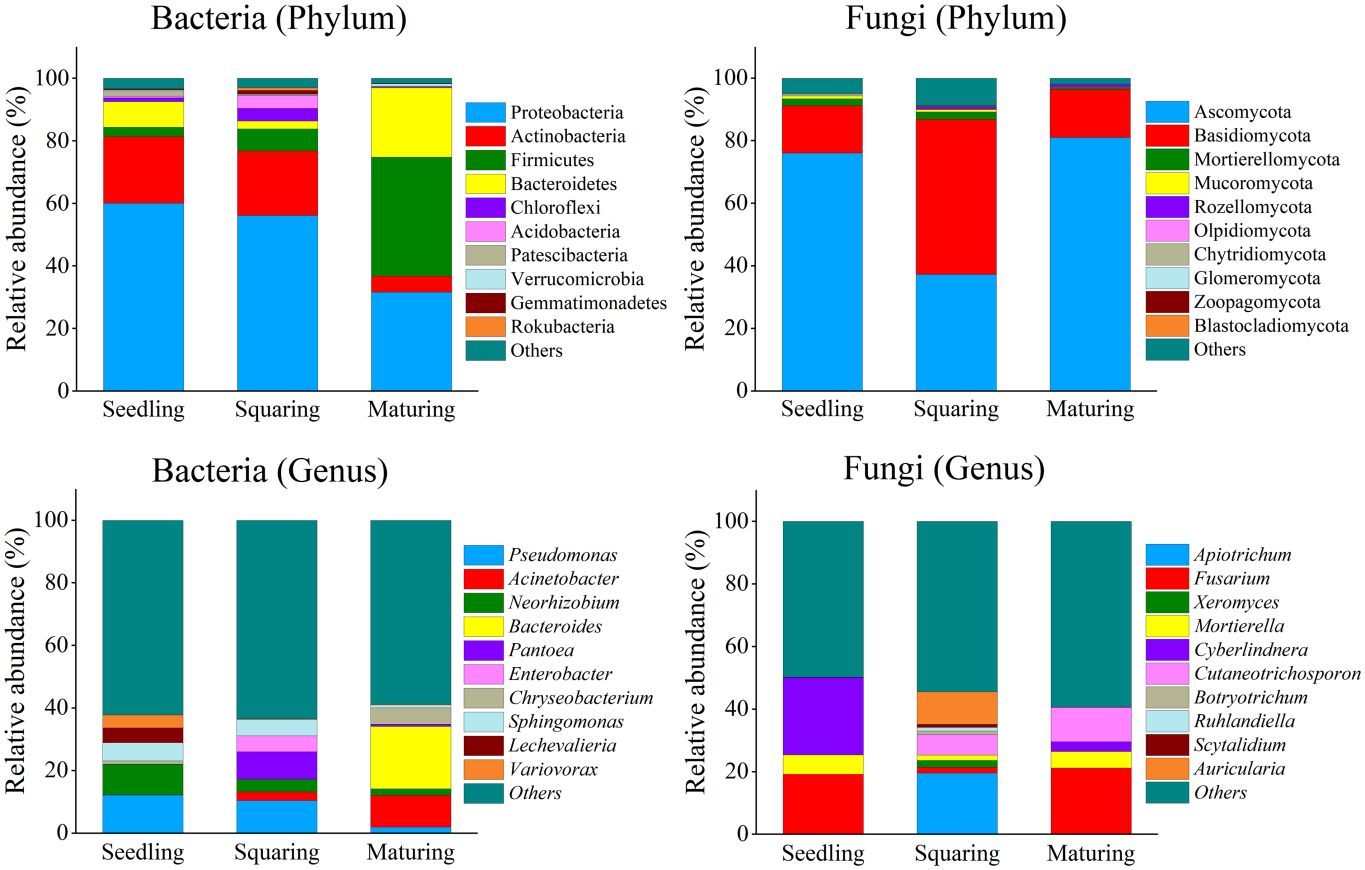
Phyllospheric bacterial and fungal community compositions at the phylum and genus levels. Taxa with relative abundances in the top 10 are shown, and each bar represents the percentage mean value of different tobacco growing stages.

Considering the microbial phyla (Figure 1), Proteobacteria and Actinobacteria massively dominated the phyllospheric bacterial communities. At maturing stage, however, their dominance was partially overcome by Firmicutes (38.05%) and Bacteroidetes (22.19%). For fungi, Ascomycota and Basidiomycota highly dominated the phyllospheric fungal community, and their proportions were apparently distinct at different growth stages. The dominance of Basidiomycota was significantly higher in squaring stage samples than in those of seedling and maturing stages, which has been accompanied with the significant minimum of Ascomycota at the squaring stage.

According to the results (Figure 1), the differences at the genus level in microbial community structures among three growth stages were significant. In bacterial communities, *Pseudomonas* (12.18%) and *Neorhizobium* (9.74%) dominated the seedling stage. At squaring stage, *Pantoea* (8.79%) and *Enterobacter* (5.07%) were dominant, and yet *Acinetobacter* (10.18%) and *Bacteroides* (19.97%) successfully invaded the phyllosphere to become the dominant bacteria at maturing stage. In addition, *Chryseobacterium* also had a significant proportion at maturing stage, accounting for 5.26%. For fungal community, *Fusarium* (19.21%) and *Cyberlindnera* (24.72%) dominated at seedling stage, while *Apiotrichum* (19.59%) and *Auricularia* (10.36%) were dominant at squaring stage. At maturing stage, however, the proportion of *Cutaneotrichosporon* increased sharply to 11.03%. Generally, the patterns change of bacterial communities at both phylum and genus levels across the growing stages of tobacco seemed to be more dynamic than those of fungi.

**Figure 2:**
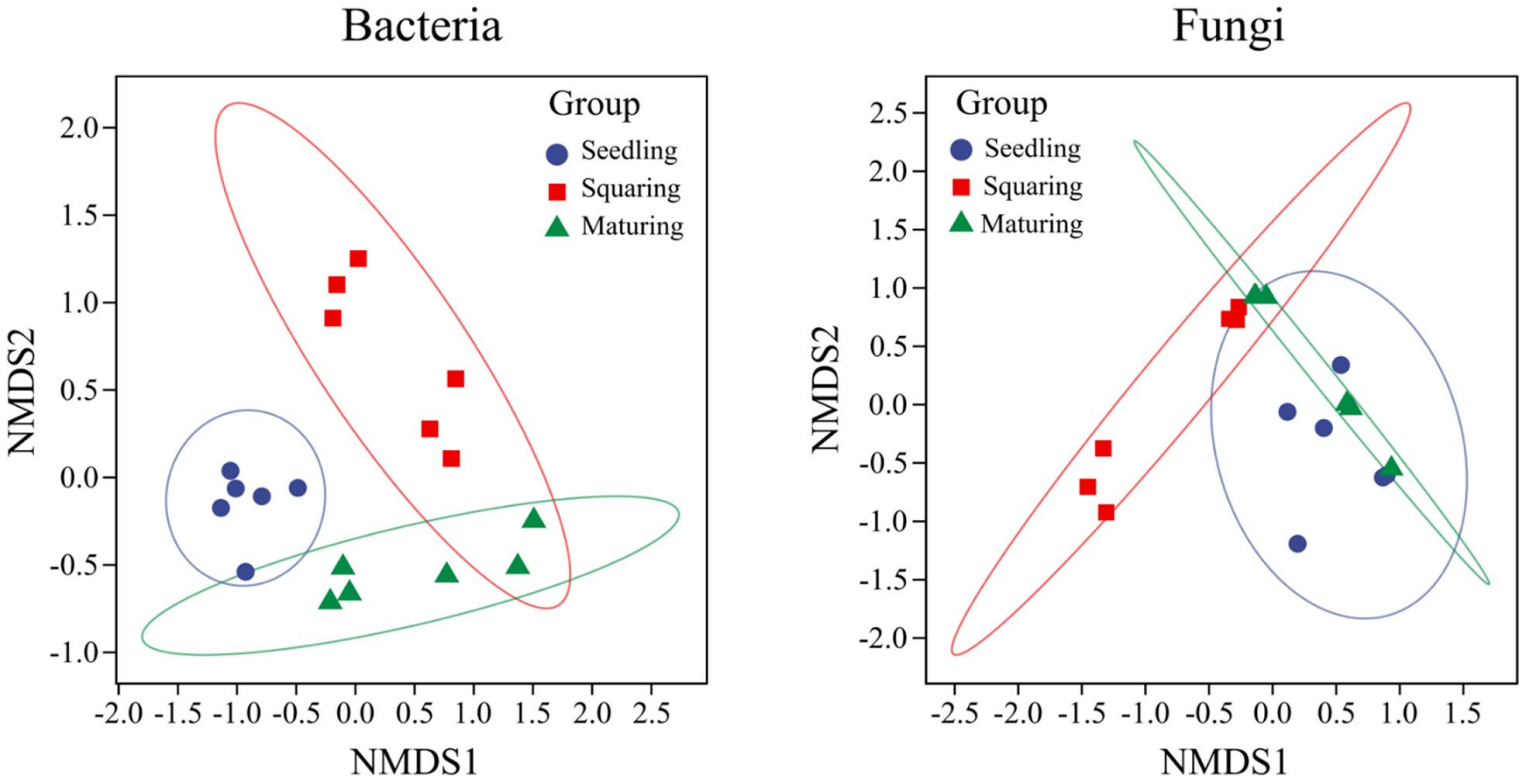
Non-metric Multi-Dimensional scaling analysis of phyllospheric bacterial and fungal communities. Different colors represent different groups (tobacco growing stages), and the distance between pairwise points indicates the degree of variation.

**Figure 3:**
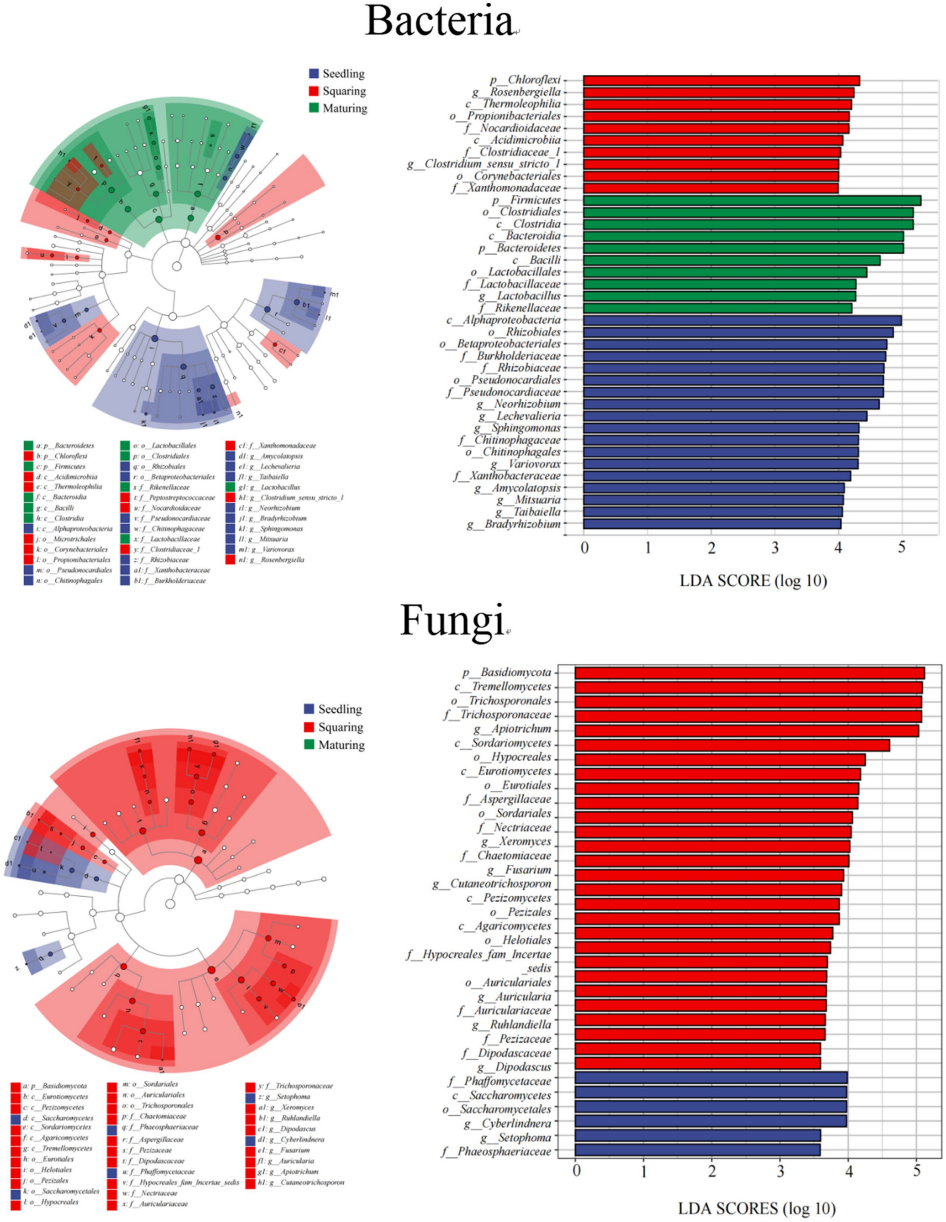
LEfSe and Cladogram of microbial community in phyllosphere (LDA scores >3.5). The lowercase letters of p, c, o, f and g represent phylum, class, order, family and genus, respectively.

As demonstrated by NMDS analysis, the overall variation pattern in microbial communities among different stages was really high, and the samples were clearly distinguished from each other (Figure 2). From seedling to maturing stage, some new taxa successfully invaded the leaf surface, causing the bacterial communities from different samples to be grouped together, but were separated from each stage (Figure 2). This pattern was further supported by the similarity analysis (ANOSIM), revealing that the phyllospheric bacterial community significantly differed (*p* < 0.01) between any two of stages compared (Table 2). However, the beta diversity of fungal community was characterized by non-significant difference (*p* > 0.05), except, between seedling and squaring stages (Figure 2; Table 2). We further used Linear discriminant analysis effect size cladogram to display the specific microorganisms (Figure 3). It is worthwhile to note that, among the bacterial microbes, 38 distinct bacterial species were found (LDA scores >3.5), of which Proteobacteria (Alphaproteobacteria) was the most key bacterium in seedling stage. Chloroflex was the most pivotal bacterium in squaring stage, and Firmicutes was the most influential bacterium in maturing stage. Within fungal community, Saccharomycetes was the most influential microbe in seedling stage, Basidiomycota (Trichosporonales) in squaring stage, while no influential fungi community was identified in maturing stage. Under similar analysis conditions, distinct bacterial species were certainly more enriched across all stages than the fungal microbes, which is exceedingly consistent with the above research results. Therefore, we inferred that, unlike the fungal microorganisms, phyllospheric bacterial microbes are more likely to discern functional profiles at different plant growth stages.

**Table 2:** Pairwise community dissimilarity test of phyllospheric bacterial and fungal communities using analysis of similarity (Anosim) based on Bray-curtis distances.

Community	Bacteria			Fungi		
	Seedling	Squaring	Maturing	Seedling	Squaring	Maturing
Seedling		0.919	0.615		0.702	0.152
Squaring	**0.004**		0.523	**0.003**		0.365
Maturing	**0.002**	**0.002**		0.123	**0.007**	

Upper diagonal cells are R values, and lower diagonal cells are *p* values. Values in bold represent that there is a significant difference (*p* < 0.05) between microbial communities for pairwise samples.

### 3.3. Functional features of microbial community

We used the PICRUSt 2 program to insightfully elucidate the vital role microorganisms play in the tobacco phyllosphere. A total of 60 (bacteria) and 29 (fungi) metabolic pathways were obtained based on microbial MetaCyc profile, which were mainly assigned to various aspects of metabolism. Although there is a similarity of phyllospheric functional features, some specific metabolic pathways were observed at different tobacco growing stages (Supplementary Table S2). For bacteria, the degradation ability of leaf bacterial microorganisms significantly decreased at maturing stage. For example, the metabolic feature of nicotinate degradation was remarkably observed down-regulated at maturing stage, unlike the seedling stage. This might have been mediated by the genera *Variovorax*, *Pseudomonas*, and *Neorhizobium*. Similarly, *Enterobacter*, Sphingomons, Rosenbergiella, and Bacillus also play important roles in various functional features. For fungi, the significant difference in functional prediction of leaf fungal microorganisms was apparent between squaring and maturing stages. Compared to the squaring stage, the functional features including L-leucine degradation, glucose and glucose-1-phosphate degradation, sucrose degradation, sulfate reduction, and monoacylglycerol metabolism were significantly decreased at maturing stage. The results are likely due to the significant difference in taxa, such as *Apiotrichum*, *Cutaneotrichosporon*, *Cyberlindnera*, *Ustilaginoidea*, *Scytalidium* and *Penicillium*.

### 3.4. The co-occurrence networks

An ecological measurement adapted from checkerboard units was conducted to further appraise the organization of the phyllospheric microbial communities. After quality filtering and OTUs grouping, we identified 2,616 (bacteria) and 2,346 (fungi) OTUs represented by no less than 5 of the 18 samples. According to the topological properties, non-random co-occurrence pattern was exhibited in phyllospheric bacterial community, whereas scale-free pattern was found in fungal community (Supplementary Figure S3).

**Figure 4:**
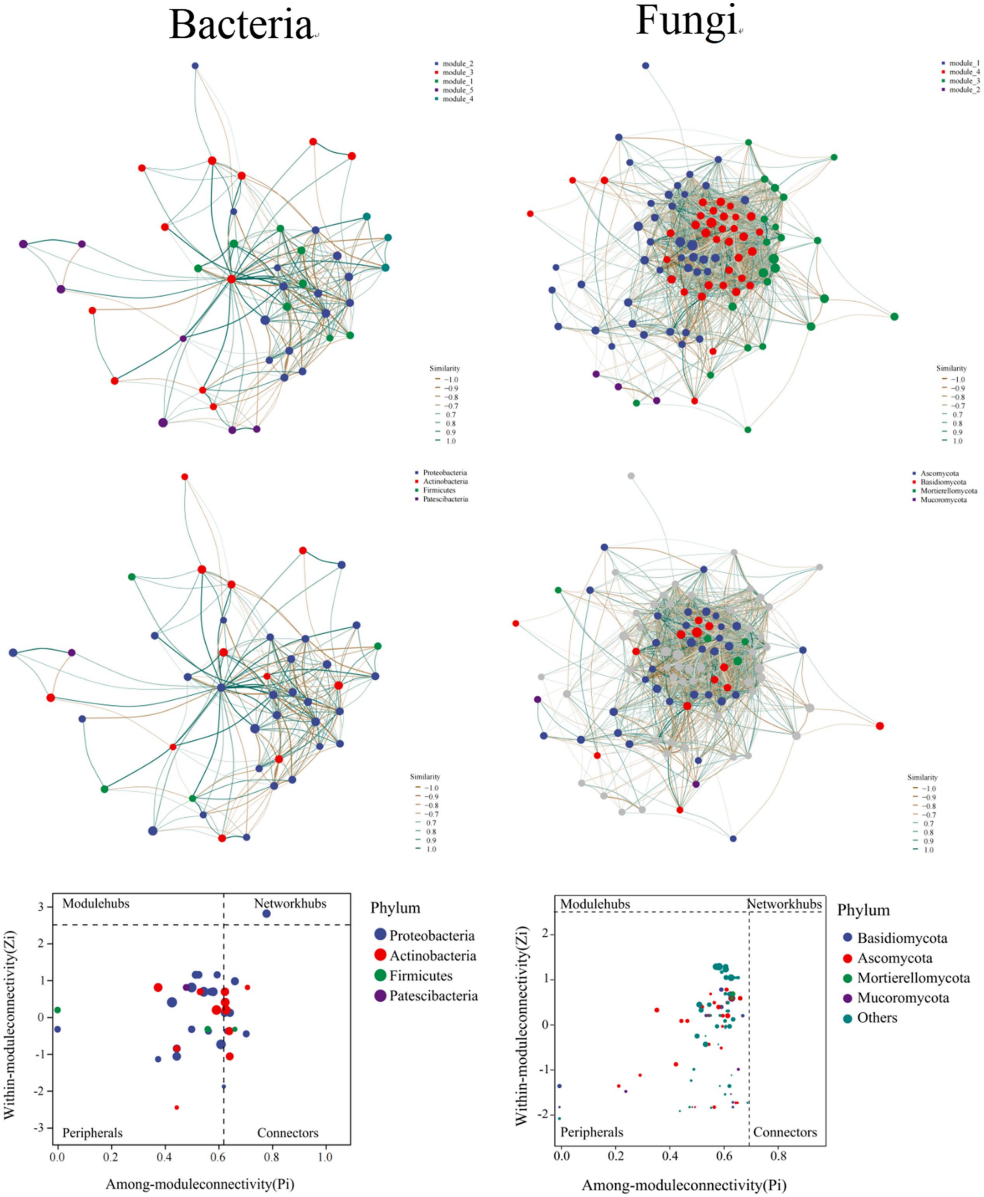
The co-occurrence network and topological roles of OTUs between bacterial and fungal communities.

Each network’s topology corroborated well with the power-law distribution. The total vertices number was 44 and 162 for the bacterial and fungal networks, respectively. The total number of edges was 280 and 5,457 for the abovementioned two networks. The values of modularity that represent these networks’ modular architecture were 0.459 and 0.529 for bacteria and fungi, respectively. To this end, the result of 5 and 4 modules were observed for the bacterial and fungal networks, respectively, (Figure 4). Meanwhile, the average nearest neighbor degree, average path length, closeness centrality, transitivity, and other estimated parameters were also disparate for these two networks (Supplementary Table S3).

Different vertices may solely play various topological roles in a network, which can be depicted using Zi-Pi plots (Figure 4). With bacterial network, 68.19% of the OTUs were assorted into peripherals, and 29.55% were classified into connectors, while only 2.27% (1 vertice) could be assorted as network hubs. All bacterial community members assorted as hubs pertained to the Proteobacteria (Gammaproteobacteria). For fungal network, however, we observed that almost OTUs were classified into peripherals, which means that OTUs in fungal network levels are lowly connected among each other.

### 3.5. The environment factors driving microbial communities

As shown by our results, the hypothesis that phyllospheric microbial communities are steadily influenced by external environment over time, and thus both micro-environmental and host factors are more likely to be involved, was strongly supported (Whipps et al., 2008). By considering to which extent the microbes are influenced by environmental factors, we took into our consideration different parameters at growing stages and divided them into two groups: leaves nutrient levels and seasonal characteristics (Table 3). From seedling to maturing stage, the concentrations of N and nicotine typically increased, but contents of sugar decreased. The elements of K and Mg had their highest concentrations at squaring stage. However, the level of Ca at squaring stage was significantly low. The difference in concentrations of Cl at different stages was not significant (*p* > 0.05). Furthermore, significant positive correlations were observed between Ca and N (*r* = 0.676, *p* < 0.01), nicotine and N (*r* = 0.689, p < 0.01), and K and Mg (*r* = 0.500, *p* < 0.05). Significant negative correlation at *p* < 0.05 and/or *p* < 0.01 was observed between the parametric pairs Mg and sugar (*r* = −0.596), K and sugar (*r* = −0.492), Cl and sugar (*r* = −0.559), and nicotine and sugar (*r* = −0.500).

**Table 3:** The environmental characteristics at different growing stages of *Nicotiana tabacum*.

	Leaves nutrient concentraations (mean ± SD)							Seasonal characteristics (monthly average)					
	N (%)	K (%)	Ca (g/kg)	Mg (g/kg)	Cl (%)	Sugar (%)	Nicotine (%)	TMP	TMN	TMX	PRE	VAP	PET
Seedling	2.4 ± 0.5^b^	2.2 ± 0.5^b^	26.0 ± 4.7^ab^	4.7 ± 0.5^b^	0.3 ± 0.1^a^	40.5 ± 2.3^a^	0.3 ± 0.3^b^	11.5	4.6	18.5	29.3	4.9	3.7
Squaring	2.5 ± 0.7^b^	3.4 ± 0.5^a^	19.9 ± 3.6^b^	6.8 ± 1.7^a^	0.4 ± 0.1^a^	31.7 ± 2.4^b^	1.7 ± 0.8^a^	18.2	14.2	22.2	270.4	15.1	2.7
Maturing	4.6 ± 0.4^a^	2.2 ± 0.5^b^	32.5 ± 8.6^a^	5.0 ± 1.3^b^	0.3 ± 0.1^a^	25.7 ± 5.0^c^	2.6 ± 1.2^a^	13.1	8.7	17.5	46.9	9.6	2
p(K-S)	0.332	0.997	0.389	0.653	0.992	0.998	0.684	0.000	0.015	0.000	0.000	0.019	0.014

Different lowercase letters, column wise, represent that the values are significant different among tobacco growing stages at *p* < 0.05, following multiple comparisons with Duncan test. p(K-S) = *p* value of Kolmogorov–Smirnov test. The TMP, mean temperature (unit: degrees celsius); TMN, mean minimum temperature (unit: degrees celsius); TMX, mean maximum temperature (unit: degrees celsius); PRE, precipitation rate (unit: mm/month); VAP, vapor pressure (unit: hPa); PET, potential evapo-transpiration (unit: mm/day).

**Figure 5:**
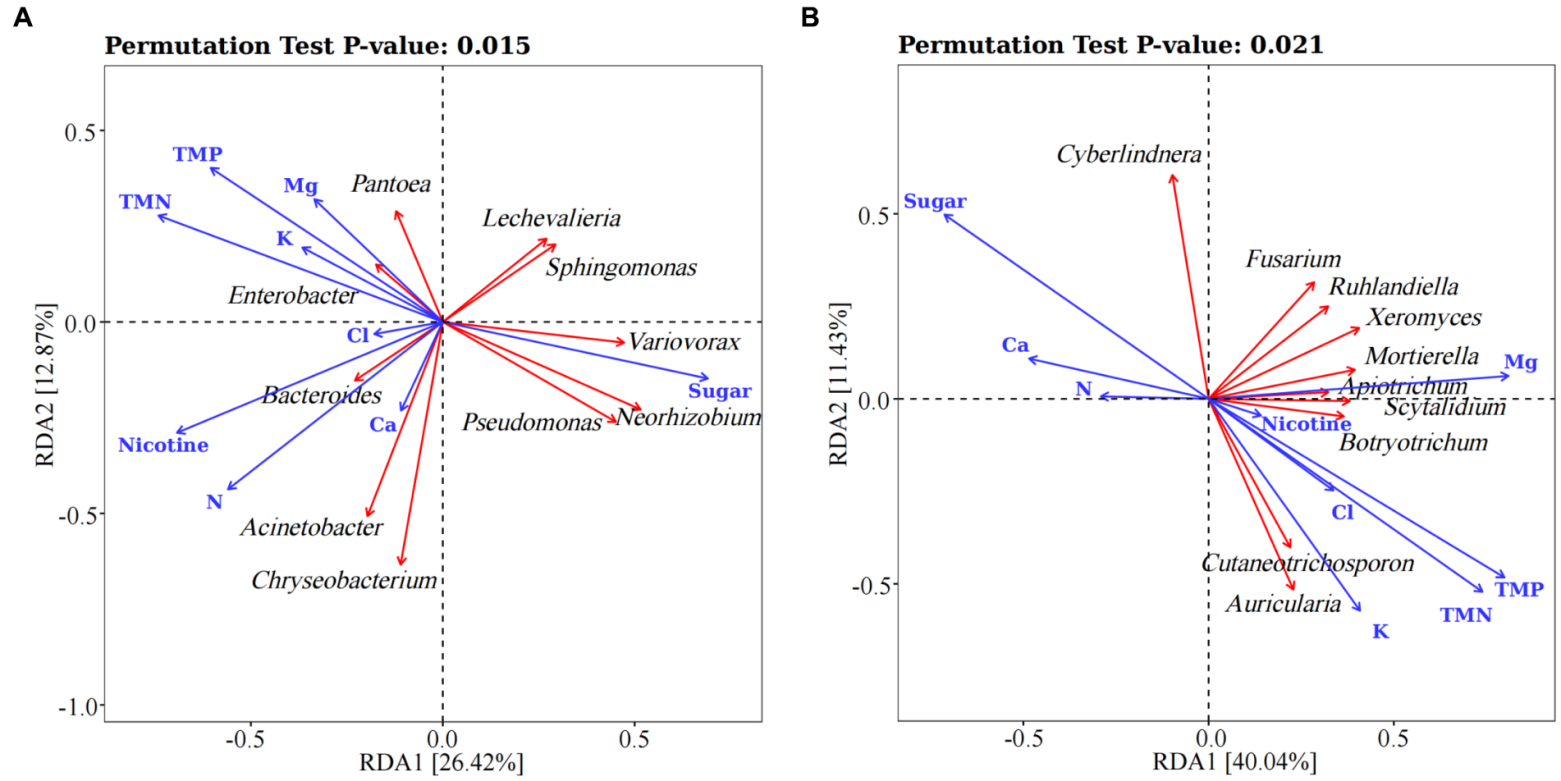
The Redundancy Analysis linking phyllospheric bacterial **(A)** and fungal communities **(B)** to their environment drivers. TMP: mean temperature (unit: degrees celsius), TMN: mean minimum temperature (unit: degrees celsius).

We used the redundancy Analysis (lengths of gradient <3) to explain changes in microbial communities by environmental factors (Figure 5). According to the Monte Carlo permutation test results, it was revealed that the concentrations of nicotine (*r*2 = 0.517, *p* < 0.01), sugar (*r*2 = 0.462, *p* < 0.01) and N (*r*2 = 0.445, *p* < 0.05) were highly correlated with the distribution of bacterial community. The bacterial genera *Neorhizobium*, *Sphingomonas*, *Lechevalieria* and *Variovorax* were mainly affected by sugar and nicotine concentrations, while *Acinetobacter* was mainly affected by N concentration (Figure 5A; Supplementary Table S4). For fungi, however, the concentrations of sugar (*r*2 = 0.645, *p* < 0.01), Mg (*r*2 = 0.475, *p* < 0.05) and K (*r*2 = 0.432, *p* < 0.05) had a considerable effect on the fungal community, particularly for the genera *Apiotrichum, Xeromyces*, *Cutaneotrichosporon*, *Auricularia* and *Cyberlindnera* (Figure 5B; Supplementary Table S4). Notably, the seasonal factors of TMP and TMN were specifically correlated with either bacterial or fungal communities.

## 4. Discussion

### 4.1. Microbial community dynamics

Phyllospheric microbial communities during plant development often exhibit distinct successions. In accordance with this, the abundance of foliar bacteria from Proteobacteria and Actinobacteria phyla decreases during tobacco development process, as they are gradually replaced by bacteria from Firmicutes and Bacteroidetes. These findings concur with those from the recent study which showed that considerable interactions exist among bacterial microorganisms, and that a pre-established community is highly against the perturbations by the next community (Gong and Xin, 2021). A direct inhibition of Firmicutes by Proteobacteria in *Arabidopsis* leaves have been affirmed. Similar results were reported by Bashir et al. (2022), which most likely revealed a pattern of almost unidirectional antibiosis. In fungal community, Ascomycota and Basidiomycota were the most dominant fungal phyla groups from phyllosphere samples, followed by Mortierellomycota, and this finding is corroborated with those of previous studies (Ma et al., 2012).

In the early stage of nutrient growth (seedling stage), bacterial alpha and beta diversities tend to have lower values (Tables 1, 2). These findings might explain, in part, that it was difficult for initial foliar microorganism upon colonization to hold on to new host plant, and they were subsequently modified by the new host plants. Nevertheless, bacterial diversities in middle stage of development (squaring stage) were significantly increased, and the bacterial communities were dominantly composed of members of *Pantoea*, *Enterobacter*, *Sphingomonas* and *Pseudomonas*. Similarly with the bacterial community, the highest fungal alpha and beta diversities were also present in squaring stage. The early stages of tobacco development are critical because the plant might be exposed to diseases and various stress conditions, hence the abundance of *Sphingomonas* and *Pseudomonas*. For instance, the squaring stage of tobacco growth is mostly influenced by high rainfall intensity in summer for most subtrobical areas in China. Our results are corroborated with those of Wang et al. (2022), who argued that the enrichment of these genera in summer might be a”cry for help” strategy of plants for the recruitment of microbes in response to biotic and abiotic stresses (Wang and Song, 2022). These heightened levels in diversity and species richness might play part in high functional redundancy within microbiome, and thus could enable it to cope with complex environmental changes and quickly recover from stress. At the late plant development (maturing stage), however, phyllospheric microbial diversities reached their minimum level, with several dominant members including, *Acinetobacter* and *Chryseobacterium* as bacteria, and the fungal microbes like *Fusarium*, *Cutaneotrichosporon* and *Mortierella*. The study of Xu et al. (2009) showed that more complex microbial communities exist during the initial reproductive growth stages of soybean than during its late stages. Ascomycota has been reported to be at the highest abundance in early and late of plant growing season, but Basidiomycota which often feature a yeast phase, were in the highest abundance in middle of the season. A possible explanation for these phenomena is that phenotypically, plant leaves differ during their different development stages, for which a batch of plant leaf migrants or colonizers favorize, hence, the disparity in microbial community composition. The host plant’s ecology has a vital role in determining foliar microbial community structure, by controlling the morphology and physiology of leaves. In addition, synergic and/or antagonistic effect between microbial cells landing on the leaf surface is also a potential mechanism for phyllosphere microbiome assembly (Faust and Raes, 2012). For example, *Sphingomonas* has transporting-related proteins that can carry sugars, vitamins and siderophores, which are the characteristics that may enable it to be highly competitive with other colonizers.

However, although plants of varied ages distinguished phyllosphere microbiome, a certain proportion of microbial taxa are “shared,” in what regarded as a conserved phenomenon (Massoni et al., 2020). Studies of microorganisms in different plant organs or phyllosphere have harbored a “core microbiome.” In our study, core bacterial microbiota during the development of plant belonged to Proteobacteria (Figure 4), with a member of the class Gammaproteobacteria, in which genera *Pseudomonas*, *Acinetobacter*, *Pantoea*, and *Enterobacter* include. Similar results were reported by Wang et al. (2022). It was reported that these hub members are considered as biocontrol bacteria and have antibiotic activity (Helfrich et al., 2018). For example, *Enterobacter* had an adverse effect on the Erwinia bacteria group during phyllosphere colonization. In various studies, the bacterial pathogen Erwinia has caused diseases with detrimental effects to a great variety of economically important plant species, like cucumber, muskmelon, tomato and tobacco (Nazareno et al., 2016). In the fungal community, no core microorganisms were found. The possible reasons could be that some fungi members colonizing the foliar surface are able to actively penetrate the leaf interior *via* stomata or other epidermal regions to establish an association between endophytic and epiphytic regions, thus causing more changeable and diverse microbial lineages (Dhayanithy et al., 2019).

### 4.2. Phyllomicrobiomes as mediating agents of ecological functions

Phyllosphere microbiota influence host plants in many ways. Of all the members of phyllosphere bacterial lineages, the Proteobacteria is often the most dominant taxa. Studies conducted by Abadi et al. (2021) have confirmed that some representative members of Proteobacteria like *Acinetobacter*, *Enterobacter*, *Pantoea*, *Pseudomonas*, Rhizobium and *Sphingomonas*, are metabolically diverse. We observed that *Pseudomonas* was sustainably survived across the whole development of tobacco leaves, followed by *Sphingomonas* and *Neorhizobium*. However, the frequency of these members fluctuates with host age, thus causing differences in some metabolic functions, such as nicotinate degradation, 2-aminophenol degradation, protocatechuate degradation, gallate degradation, and methylgallate degradation, etc. (Supplementary Table S2). In addition, *Variovorax* was found to be popular on phyllosphere as well, and it was more likely to harbor similar ecological functions with common genera of Proteobacteria, which is in agreement with previous studies (Crombie et al., 2018; Palmer et al., 2021). These studies corroborated the idea that the functional redundancy of microorganisms seem to be a conservative phenomenon of plants.

*Enterobacter* favorably maintained an advantage on the tobacco phyllosphere during the squaring development, and thus mediated significantly different metabolic functions, like carrying out the process of 3-phenylpropanoate and 3-(3-hydroxyphenyl) propanoate degradation, cinnamate and 3-hydroxycinnamate degradation to 2-oxopent-4-enoate, and aerobactin biosynthesis (Supplementary Table S2). Similarly, studies have showed that some of *Enterobacter* species are capable to solubilize phosphate and/or potassium necessary for plant growth and development (Joshi et al., 2023). They produce plant-growth regulators or establish quorum sensing systems on leaves, which enable them to hold in check pathogens on leaves (Przemieniecki et al., 2019). Furthermore, several specific taxa such as Proteobacteria (Azoarcus) and Actinobacteria (Mycobacterium) have also been found at squaring stage, which rendered to them diverse functions for biphenyl degradation and p-cumate degradation.

However, fungal microorganisms could be essential in ecological adaptability of plant phyllosphere. The dominant taxa *Apiotrichum*, *Cutaneotrichosporon*, *Cyberlindnera*, *Scytalidium*, *Penicillium* and *Ustilaginoidea* in tobacco foliar surface could regulate key metabolic functions, such as sulfate reduction, L-leucine degradation, glucose and glucose-1-phosphate degradation, sucrose degradation, and monoacylglycerol metabolism, etc. (Supplementary Table S2). Furthermore, other phyllosphere fungi are known to be dormant saprotrophs, which have a profound effect on decomposition and nutrient cycling following leaf senescence (Poosakkannu et al., 2017).

### 4.3. Drivers of microbial community structure

Phyllosphere hyperdiverse microbial communities are related to the host’s specific functional characteristics (Rosado et al., 2018). A growing body of research suggests that the availability of carbon is the most significant determinant of their existence in the host phyllosphere. Studies of Mercier and Lindow (2000) showed that leaf exudate sugars, serving as carbon source, determine the total bacterial population size in the phyllosphere. This is in line with the results of our study that the diversity of microbial communities in the phyllosphere decreased with the change in sugar content, especially the abundance of bacterial genera *Neorhizobium*, *Sphingomonas*, *Lechevalieria* and *Variovorax*. However, it is worthy to note that the changes of these epiphytic bacteria seem to ultimately define the microbial community structure, through their antimicrobial activities or the competition for nutrients among other microbes. For example, the foliar *Sphingomonas* in *Arabidopsis* can directly compete with plant pathogen *Pseudomonas syringae* for sugars like fructose, glucose and sucrose, and limits its growth (Innerebner et al., 2011).

Phyllosphere microbial ecosystem was also highly shaped by plant N, altering the total microflora counts and species composition. The previous study demonstrated that more abundant bacteria in the phyllosphere are proportional to the lower leaf nitrogen concentrations (Laforest-Lapointe et al., 2016), which is similar to our study findings (Tables 1, 3). High leaf nitrogen content contributes to high likelihood of a disease, which could be probably explained by nitrogen-induced pathogen susceptibility (Huang et al., 2017). In our study, however, the nitrogen content of tobacco leaves was negatively, yet significantly, correlated with some pathogenic fungi, particularly, *Fusarium* and *Botryotrichum*. The possible reason is that the effect of N on the microbial communities was not consistent and likely stage dependent. Moreover, potassium starvation response also influences representatives of some taxa of the phyllosphere microbial community. In our study, positive and significant correlations between leaf potassium content and various fungal microorganisms were observed, particularly the genera *Apiotrichum, Xeromyces* and *Cutaneotrichosporon* (Supplementary Table S4).

Nonetheless, some microorganisms in this study seemed to be undetectable in tobacco leaves. The survival of these microbes might illustrate the generally accepted principle that they were driven by different constraints of climate and dispersal. Hellberg and Chu (2015) have predicted climate-induced factors, with trends, that will affect the persistence and dispersal of phyllospheric microorganisms in myriad of ways. In our study, air temperature is considered as one of the most climatic factors influencing both bacterial and fungal communities. However, it has been argued about that the influence of leaf temperature seem to typically exceed that of air temperature, although significant variation occurs among plant species and among leaves of the same plant. Temperature on the leaf surface induces strong temporal variability in phyllosphere microorganisms, as a result of transient phenomena, diurnal cycles, and other unpredictable meteorological events (Chelle, 2005). This might confer a selective advantage to the microbial survival, especially for individual environmental microorganisms. In addition, Alsanius et al. (2017) have documented about fungal microorganisms, as they may directly be affected by the physical properties of a certain light source, whereas bacteria are likely to be influenced indirectly, through alteration of plant development process caused by various light sources.

## 5. Conclusion

Tobacco phyllospheric microbia is a dynamic process controlled by different microorganisms, specified at different development stages of the plant. Proteobacteria play a significant role in unique co-occurrence net-work features for bacterial communities, and consequently crucial in resisting external interference. We also found that the contents of sugar, nicotine, nitrogen and potassium in tobacco leaves enormously affected phyllospheric microbial dynamics during plant development, and leaf temperature mediated by air temperature is also at play. In general, we provide the evidence that tobacco phyllosphere is the resultant of a dynamic cross-kingdom succession, and bacterial community is a greater succession and more interacting microbial community than fungi.

## Data availability statement

The data presented in this study have been deposited in the NCBI database, accession numbers PRJNA989749 (bacteria) and PRJNA989756 (fungi).

## Author contributions

JG and EU: conceptualization. JG: methodology, software, validation, formal analysis, investigation, resources, data curation, writing–original draft preparation, and visualization. EU: writing–review and editing. DZ: supervision, project administration, and funding acquisition. All authors have read and agreed to the published version of the manuscript.

## Funding

This study was financially supported by the project of advanced techniques for microbial potassium fertilizer production and utilization in tobacco farming soils (grant number: SCYC202105), and Research and Innovation Team for Heritage Protection and Tourism Development (grant number: KYCXTD2023-2).

## Conflict of interest

The authors declare that the research was conducted in the absence of any commercial or financial relationships that could be construed as a potential conflict of interest.

## Publisher’s note

All claims expressed in this article are solely those of the authors and do not necessarily represent those of their affiliated organizations, or those of the publisher, the editors and the reviewers. Any product that may be evaluated in this article, or claim that may be made by its manufacturer, is not guaranteed or endorsed by the publisher.

## Footnotes

^1^http://climexp.knmi.nl/

^2^https://metacyc.org/

^3^https://www.genescloud.cn/home
