# Supplementary material for: Microbial composition and diversity of the tobacco leaf phyllosphere during plant development
Source: Front Microbiol. 2023 Jul 12;14:1199241. doi: 10.3389/fmicb.2023.1199241 (PMC10368876; doi:10.3389/fmicb.2023.1199241)
Supplement: Supplementary file 1 [file Data_Sheet_1.pdf]

*Supplementary Material*

# Microbial composition and diversity of the tobacco leaf phyllosphere during plant development

**Jianing Gao, Ernest Uwiringiyimana, Dan Zhang**

*** Correspondence:** Corresponding Author: gaozegl@163.com

## 1 Supplementary Figures and Tables

### 1.1 Supplementary Figures

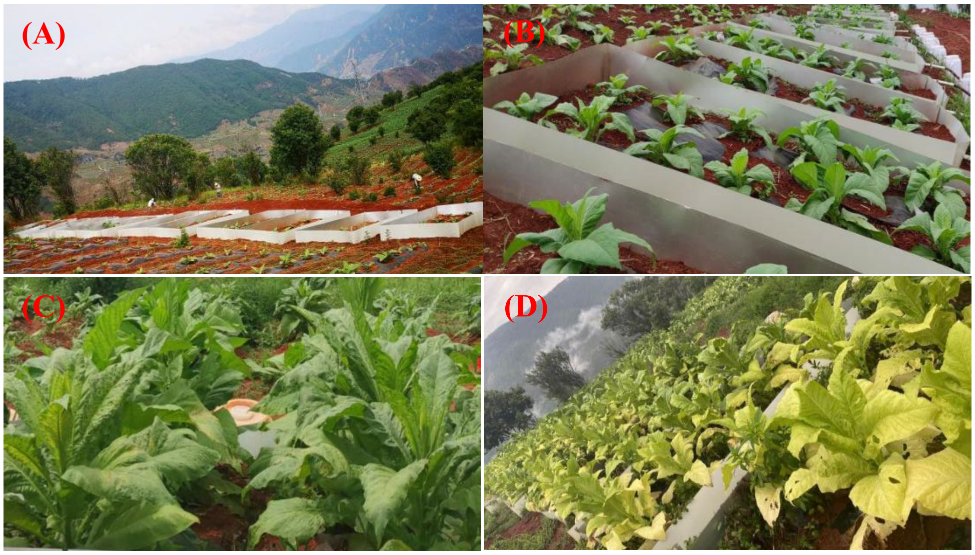


**Supplementary Figure 1.** Experimental pool and different tobacco growth stages at which samples were taken (A): experimental pool, (B): Seedling stage (April), (C): Squaring stage (July), (D): Maturing stage (October).

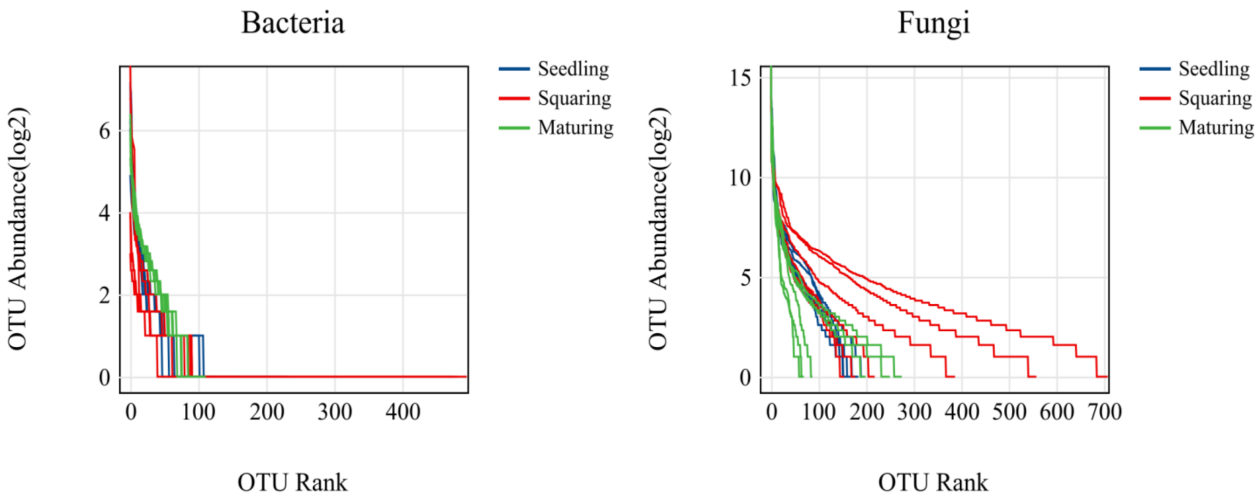


**Supplementary Figure 2.** The rank-abundance curve of bacterial and fungal communities.

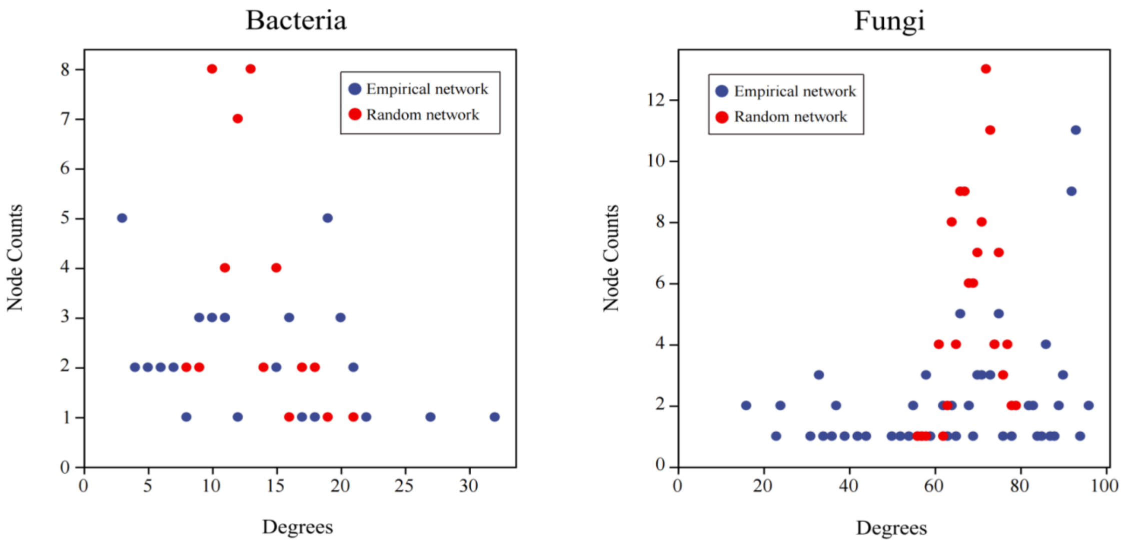


**Supplementary Figure 3.** The degree distribution of bacterial and fungal communities.

## 1.2 Supplementary Tables

**Supplementary Table 1** Sequencing data and OTU numbers of 6S rRNA and ITS genes

| Stage | | Bacteria | | | Fungi | | |
|---|---|---|---|---|---|---|---|
| | | Reads | After nonsingleton | OTUs | Reads | After nonsingleton | OTUs |
| Seedling | Range | 71079 - 105144 | 60892 - 91537 | 85 - 234 | 126516 - 143204 | 75907 - 105540 | 157 - 195 |
| | Mean | 87796 | 73989 | 154 | 132648 | 86740 | 174 |
| Squaring | Range | 79734 - 128964 | 26384 - 106317 | 70 - 496 | 96451 - 134911 | 92666 - 121901 | 155 - 709 |
| | Mean | 100722 | 63498 | 295 | 114936 | 104767 | 368 |
| Maturing | Range | 104756 - 128795 | 87614 - 106173 | 76 - 112 | 125424 - 137963 | 81418 - 123964 | 66 - 276 |
| | Mean | 113081 | 96220 | 95 | 131499 | 102463 | 159 |
| | Total | 1809586 | 1402238 | 3265 | 2274495 | 1763817 | 4205 |

**Supplementary Table 2** Analysis on the significance of the difference in prediction function of phyllospheric microbial community.

| Group | Pathway | Description | Log FC | P values | Community compositions | |
|---|---|---|---|---|---|---|
| | | | | | Phylum | Genus |
| Bacteria Squaring( Seedling) | PWY-7295 | L-arabinose degradation IV | -1.95 | 0.005 | Proteobacteria | *Herbaspirillum*, *Polynucleobacter* |
| | PWY5F9-12 | Biphenyl degradation | 0.52 | 0.010 | Proteobacteria | *Azoarcus* |
| | PWY-7209 | Superpathway of pyrimidine ribonucleosides degradation | 0.67 | 0.011 | Proteobacteria | *Nannocystaceae*, *Nannocystis* |
| | PWY-2221 | Entner-Doudoroff pathway III (semi-phosphorylative) | 2.00 | 0.000 | Actinobacteria | *Solirubrobacter* |
| | PWY-5266 (5273) | P-cumate degradation | 3.29 | 0.000 | Actinobacteria | *Mycobacterium* |
| Bacteria Maturing( Seedling) | PWY-722 | Nicotinate degradation I | -3.42 | 0.000 | Proteobacteria | *Variovorax*, *Pseudomonas*, *Neorhizobium* |
| | PWY-6210 | 2-aminophenol degradation | -2.69 | 0.044 | Proteobacteria | *Sphingomons* |
| | P184-PWY | Protocatechuate degradation I (meta-cleavage pathway) | -2.34 | 0.030 | Proteobacteria | *Sphingomonas* |
| | GALLATE-DEGRADATION-I-PWY | Gallate degradation II | -2.02 | 0.013 | Proteobacteria | *Variovorax*, *Sphingomonas*, *Pseudomonas* |
| | METHYLGALLATE-DEGRADATION-PWY | Methylgallate degradation | -1.96 | 0.013 | Proteobacteria | *Sphingomonas*, *Enterobacter*, *Pseudomonas* |
| | PWY-6895 | Superpathway of thiamin diphosphate biosynthesis II | 1.53 | 0.019 | Proteobacteria, Firmicutes | *Enterobacter*, *Rosenbergiella*, *Escherichia-Shigella* |
| | PWY-6891 | Thiazole biosynthesis II | 2.14 | 0.007 | Proteobacteria, Firmicutes | *Enterobacter*, *Rosenbergiella*, *Bacillus* |
| | THREOCAT-PWY | Superpathway of L-threonine metabolism | 2.43 | 0.004 | Proteobacteria | *Enterobacter*, *Klebsiella* |
| | PWY-6629 | Superpathway of L-tryptophan biosynthesis | 3.52 | 0.000 | Proteobacteria | *Enterobacter* |
| | PWY-7210 | Pyrimidine deoxyribonucleotides biosynthesis from CTP | 3.53 | 0.007 | Chloroflexi | *JG30-KF-CM66* |
| | LPSSYN-PWY | Superpathway of lipopolysaccharide biosynthesis | 3.58 | 0.000 | Proteobacteria | *Enterobacter*, *Arsenophonus* |
| Bacteria Maturing( Squaring) | HCAMHPDEG-PWY | 3-phenylpropanoate and 3-(3-hydroxyphenyl)propanoate degradation to 2-oxopent-4-enoate | -2.02 | 0.024 | Proteobacteria | *Enterobacter* |

| | | | | | | |
|---|---|---|---|---|---|---|
| | PWY-6690 | Cinnamate and 3-hydroxycinnamate degradation to 2-oxopent-4-enoate | -2.02 | 0.024 | Proteobacteria | *Enterobacter* |
| | PWY0-1277 | 3-phenylpropanoate and 3-(3-hydroxyphenyl)propanoate degradation | -1.70 | 0.008 | Proteobacteria | *Enterobacter* |
| | PWY-2221 | Entner-Doudoroff pathway III (semi-phosphorylative) | -1.53 | 0.006 | Actinobacteria | *Solirubrobacter* |
| | PWY-1541 | Superpathway of taurine degradation | -1.37 | 0.038 | Proteobacteria | *Paracoccus*, *Amaricoccus* |
| | AEROBACTINSYN-PWY | Aerobactin biosynthesis | -1.08 | 0.000 | Proteobacteria | *Enterobacter*, *Raoultella* |
| | CENTBENZCOA-PWY | Benzoyl-CoA degradation II (anaerobic) | -0.46 | 0.008 | Proteobacteria | *Azoarcus*, *Thauera*, *Aquicella* |
| | PWY-7274 | D-cycloserine biosynthesis | -0.38 | 0.045 | Proteobacteria | *Burkholderiaceae* |
| Fungi Maturing(Squaring) | LEU-DEG2-PWY | L-leucine degradation I | -4.01 | 0.002 | Ascomycota, Basidiomycota | *Apiotrichum*, *Cutaneotrichosporon*, *Scytalidium* |
| | GLUCOSE1PMETAB-PWY | Glucose and glucose-1-phosphate degradation | -3.85 | 0.009 | Ascomycota, Basidiomycota | *Cutaneotrichosporon*, *Penicillium*, *Ustilaginoidea* |
| | PWY-7420 | Monoacylglycerol metabolism | -3.82 | 0.009 | Ascomycota, Basidiomycota | *Cutaneotrichosporon*, *Ustilaginoidea*, *Acremonium* |
| | PWY-7268 (7269) | NAD/NADP-NADH/NADPH cytosolic interconversion | -3.79 | 0.009 | Ascomycota, Basidiomycota | *Cutaneotrichosporon*, *Ustilaginoidea*, *Acremonium* |
| | PWY-621 | Sucrose degradation III (sucrose invertase) | -3.66 | 0.010 | Ascomycota, Basidiomycota | *Cutaneotrichosporon*, *Penicillium*, *Ustilaginoidea* |
| | SO4ASSIM-PWY | Sulfate reduction I (assimilatory) | -4.13 | 0.020 | Ascomycota, Basidiomycota | *Apiotrichum*, *Cutaneotrichosporon*, *Cyberlindnera* |

Note: If Log FC>0, it is significantly up-regulated; otherwise, it is significantly down-regulated. The group in parentheses represents the control group.

**Supplementary Table 3** The topological parameters of bacterial and fungal networks.

| Topological parameters | Bacterial network | Fungal network |
|---|---|---|
| Average nearest neighbor degree | 17.052 | 87.733 |
| Average path length | 1.790 | 1.603 |
| Betweenness centrality | 13144.0 | 18134.8 |
| Closeness centrality | 10.047 | 22.975 |
| Degree assortativity | -0.019 | 0.110 |
| Degree centralization | 848 | 7878 |
| Density | 0.296 | 0.418 |
| Diameter | 2.610 | 1.442 |
| Transitivity | 0.612 | 0.682 |

**Supplementary Table 4** Spearman correlation analysis for relative abundance of microbial community (Top 10) at the genus level and phytochemical characteristics from leaves of of *Nicotiana tabacum.*

| Community | Genus level | N | Ca | Mg | K | Cl | Sugar | Nicotine |
|---|---|---|---|---|---|---|---|---|
| Bacteria | *Pseudomonas* | -0.148 | -0.128 | 0.051 | 0.040 | 0.011 | 0.091 | -0.351 |
| | *Acinetobacter* | 0.545* | 0.186 | 0.356 | 0.016 | -0.067 | -0.318 | 0.426 |
| | *Neorhizobium* | -0.299 | 0.093 | -0.294 | -0.255 | -0.185 | 0.615** | -0.607** |
| | *Bacteroides* | 0.332 | 0.422 | -0.214 | -0.186 | -0.053 | 0.109 | 0.246 |
| | *Pantoea* | 0.223 | -0.177 | 0.479* | 0.370 | 0.599** | -0.600** | 0.487* |
| | *Enterobacter* | 0.079 | -0.149 | 0.414 | 0.398 | 0.096 | -0.311 | 0.178 |
| | *Chryseobacterium* | 0.203 | 0.211 | -0.040 | -0.059 | 0.124 | 0.225 | -0.180 |
| | *Sphingomonas* | -0.216 | 0.095 | -0.400 | -0.457 | -0.395 | 0.661** | -0.498* |
| | *Lechevalieria* | -0.448 | -0.114 | -0.392 | -0.172 | -0.238 | 0.659** | -0.690** |
| | *Variovorax* | -0.411 | 0.0140 | -0.333 | -0.354 | -0.180 | 0.703** | -0.728** |
| Fungi | *Apiotrichum* | -0.321 | -0.438 | 0.606** | 0.744** | 0.504* | -0.758** | 0.431 |
| | *Fusarium* | -0.579* | -0.342 | 0.028 | 0.148 | 0.115 | 0.119 | -0.337 |
| | *Xeromyces* | -0.153 | -0.289 | 0.498* | 0.519* | 0.312 | -0.542* | 0.370 |
| | *Mortierella* | -0.284 | -0.166 | 0.353 | 0.198 | 0.156 | 0.001 | -0.191 |
| | *Cyberlindnera* | -0.179 | 0.135 | -0.555* | -0.564* | -0.305 | 0.799** | -0.622** |
| | *Cutaneotrichosporon* | -0.303 | -0.522* | 0.435 | 0.591** | 0.318 | -0.741** | 0.381 |
| | *Botryotrichum* | -0.608** | -0.429 | 0.262 | 0.365 | 0.009 | -0.241 | -0.191 |
| | *Ruhlandiella* | -0.329 | -0.389 | 0.545* | 0.383 | 0.173 | -0.418 | 0.157 |
| | *Scytalidium* | -0.341 | -0.351 | 0.458 | 0.414 | 0.241 | -0.235 | -0.034 |
| | *Auricularia* | -0.309 | -0.572* | 0.649** | 0.799** | 0.438 | -0.757** | 0.346 |
